# Testing the effectiveness of combined attention modification training with right dorso-lateral prefrontal cortex theta-burst stimulation on reducing levels of anxiety and attentional bias

**DOI:** 10.1007/s00221-025-07040-9

**Published:** 2025-05-04

**Authors:** Maria Sikki, Katerina Konikkou, Nikos Kostantinou, Kostas A. Fanti

**Affiliations:** 1 Present Address: University of Cyprus, Nicosia, Cyprus; 2 Cyprus University of Technology, Nicosia, Cyprus

## Abstract

Neurostimulation techniques, such as continuous theta-burst stimulation (cTBS), over the dorsolateral prefrontal cortex (DLPFC) have been associated with improvements in anxiety symptoms and emotion processing. The aim of this feasibility study was the evaluation of the effectiveness of cTBS over the right DLPFC combined with Attention Modification Training (AMT) on reducing levels of anxiety and attentional bias. A 40s-cTBS session (real or sham) over the right DLPFC was administrated at 8 treatment sessions over a 2-week period, and each cTBS treatment was followed by computer-based AMT (real or control). Eighty-nine participants (*M*age = 21.29, *SD* = 2.06, 50.56% females) differentiated on levels of anxiety were randomly assigned to the following treatment groups (i) cTBS and AMT, (ii) cTBS and control AMT, and (iii) sham cTBS and AMT. Findings suggested that cTBS combined with AMT treatment was not superior to sham cTBS and AMT on reducing self-reported anxiety symptoms. However, combined cTBS and AMT was associated with increased attention towards positive stimuli and increased gaze fixation in the mouth region of happy facial expressions. Current results provide promising evidence for the effectiveness of AMT in reducing anxiety symptoms and contribute to existing knowledge on how inhibitory stimulation over the right DLPFC combined with AMT may influence emotion processing. Present findings can inform future treatments designed to address the attention mechanisms leading to anxiety symptoms.

**Supplementary Information:**

The online version contains supplementary material available at 10.1007/s00221-025-07040-9.

## Introduction

Pharmacotherapy and cognitive behavior therapy are among the most empirically supported forms of treatment for anxiety disorders; however, many patients do not achieve remission status (Bystritsky 2006; Springer et al. 2018; Strawn et al. 2018). The great variability in treatment outcomes in anxiety has led to the development of biologically or experimentally informed interventions. Regardless of the type of anxiety disorder, anxious individuals exhibit attention bias for threatening stimuli as well as emotion dysregulation (Amir et al. 2009; Bar-Haim et al. 2007; Mennin et al. 2005), which have been associated with deficient prefrontal functioning (Chavanne and Robinson 2021; Kenwood et al. 2022). Investigating the synergetic action between brain and attention deficits can enhance the understanding of emotional problems associated with anxiety and inform effective interventions. Hence, the current feasibility study aims to combine two promising treatments that target the attentional and emotional impairments leading to anxiety: continuous Theta Burst Stimulation (cTBS) and Attention Modification Training (AMT).

### Transcranial magnetic stimulation

Non-invasive brain stimulation techniques have been largely successful in the treatment of anxiety disorders (Cirillo et al. 2019; Moreno et al. 2021; Sagliano et al. 2019; Vicario et al. 2019). Initial evidence suggests that Theta Burst Stimulation (TBS), which is a novel Transcranial Magnetic Stimulation protocol that stimulates local regions of the cortex with a very short application period, is also successful in reducing symptoms associated with anxiety disorders (Li et al. 2022; Zhang et al. 2023). Whilst ordinary repetitive Transcranial Magnetic Stimulation requires approximately 30 min to be fully effective (e.g., low stimulation at 1 Hz; Maeda et al. 2000), TBS protocols need between 20s and 3 min (Lowe et al. 2018). One of TBS main stimulation paradigms is **continuous theta burst stimulation (cTBS)**. The physiological mechanisms behind cTBS are theorized to be analogous to long-term synaptic depression (Huang et al. 2011; Suppa et al. 2016), and cTBS can create neuronal inhibition for up to 50–60 min (Huang et al. 2005; Wischnewski and Schutter 2015). This stimulation protocol is a promising therapeutic tool for mental health disorders, and cTBS is considered safe, with limited side effects (Rossi et al. 2020).

The right dorsolateral prefrontal cortex (DLPFC) is a common stimulation site in neural studies, as functional neuroimaging research have shown hyperactivation of this area among anxious individuals (Bishop et al. 2004; Fu et al. 2017) as well as associations with top-down emotional attention deficits (Andreescu et al. 2015; Sarter et al. 2001). In addition, dysregulation in the dorsolateral prefrontal cortex (DLPFC) has been implicated in the attentional biases that are evident in anxiety disorders (Valadez et al. 2022). Specifically, hypoactivity in the left DLPFC is associated with reduced approach-related behaviors, while hyperactivity in the right DLPFC corresponds to heightened vigilance toward threat (Zwanzger et al. 2014). Modulating activity in these regions can influence attentional control and emotional processing (Madonna et al. 2019; Zwanzger et al. 2014). When applied to the right DLPFC, we expect cTBS to diminish hyperactive threat-related processing, thereby rebalancing neural activity toward a more adaptive state. This reduction in threat-focused processing may allow greater allocation of attentional resources to non-threatening, positive stimuli, such as happy facial expressions (Konikkou et al. 2020). Although various findings point to decreases in self-report anxiety after neurostimulation in DLPFC (Diefenbach et al. 2013, 2016; Dilkov et al. 2017; Schutter et al. 2001; White and Tavakoli 2015), only limited studies have used cTBS protocols with promising results (Li et al. 2022; Zhang et al. 2023). By stimulating the right DLPFC region, we aimed to alter cognitive functions that interfere with anxiety, which might influence emotional and attentional deficits through neural pathways connecting prefrontal with limbic brain regions (i.e., amygdala; Ochsner and Gross 2007; Vuilleumier 2005). Taken together, the promising findings of stimulation studies targeting the right DLPFC in anxiety and the effectiveness of the time-efficient cTBS brain stimulation protocol, suggest that it is worth exploring further for therapeutic applications (Li et al. 2022; Zhang et al. 2023), an aim of the current study.

Importantly, effects in cognitive functions and attention processes of emotional stimuli have also been found following cTBS. A comprehensive review (Ngetich et al. 2020) concluded that even though cTBS over the right DLPFC leads to impaired attention, it benefits other cognitive functions by reducing the effect of distractors. For example, in the case of decision making, cTBS over the right DLPFC is associated with beneficial effects by reducing impulsivity and inducing participants to favor large but delayed rewards instead of immediate but small rewards (Cho et al. 2010, 2012). Evidence for the therapeutic effects of TBS protocols over the right DLPFC on emotional attention comes from the work of Cao and colleagues (2018), who reported increased brain-level activation to happy faces, as recorded by electroencephalogram activity on the scalp during an emotion recognition Go/NoGo task. Increased brain activity after right DLPFC cTBS was also reported by Keuper et al. (2018), but within the occipital-parietal brain area in response to negative pictures. Additional work provided experimental confirmation that inhibitory cTBS over the DLPFC leads to increased attention facilitation to positive stimuli in healthy participants (Konikkou et al. 2020). These effects of cTBS on emotional processing confirm previous evidence for improvement in emotional recognition accuracy of facial expressions after bilateral cTBS over the DLPFC (Konikkou et al. 2020). We aim to apply cTBS among individuals differentiated on levels of anxiety.

### Attention modification training (AMT)

Cognitive changes are a core feature of anxiety, including disruptions in attention, inhibitory control, and regulation of autonomic arousal (Kenwood et al. 2022). Changes in attention allocation can take the form of selective attention toward threatening cues, indicating hypervigilance (Richards et al. 2014), or bias away from threatening cues, an indication of avoidance (Koster et al. 2006). It has been suggested that attention bias is not only a fundamental phenotype of anxiety disorders but might also lead to the preservation of the problem (Mogg and Bradley 2016). Because of the strong evidence of attention bias deficits in anxiety disorders, we chose to combine non-invasive brain stimulation with AMT.

AMT has been mostly used in anxiety and attention deficits studies (De Voogd et al. 2016; Hakamata et al. 2010). The aim of AMT paradigms is the reduction of negative attentional bias by training people to disengage from negative valence cues using training versions of attention tasks. Of these, the visual dot-probe task is one of the most frequently used tasks to assess attention bias in anxiety (MacLeod and Mathews 2012). In the assessment version of this task, probes are presented equally often in the screen locations, associated with either disorder-relevant (i.e., threatening or happy stimuli) or neutral stimuli. However, in the attention bias modification version of the task, the probes always appear in the location of the disorder-relevant stimuli (attend training) or the neutral stimuli (avoidance training). By directing attention towards the disorder-relevant stimuli bottom-up attention processes are used. Bottom-up attention refers to the attentional guidance purely by externally driven factors to stimuli that are salient because of their inherent properties relative to the background (Katsuki and Constantinidis 2014).

Additional AMT protocols seek to increase attention toward positive cues by adding visual detection trials (e.g., Corman et al. 2020; Mogg et al. 2017; Dandeneau et al. 2007; Taylor et al. 2011). This task often involves repeatedly asking the participant to find a target disorder-incompatible stimulus (e.g., a smiling face) among distracting disorder-relevant stimuli (e.g., fearful faces). Theoretically, through repetitive practice, anxious individuals indirectly learn to overcome their tendency to preferentially process disorder-relevant stimuli by using top-down attention processes (e.g., Corman et al. 2020; Taylor et al. 2011). Top-down attention refers to internal guidance of attention based on prior knowledge, willful plans, and current goals (Katsuki and Constantinidis 2014). This attentional training toward positive emotional stimuli is based on evidence that anxious individuals are characterized by avoidance towards positive information, which increases the likelihood of emotional deficits and stressful reactions (Carl et al. 2013). Studies reviewing the efficacy of attention training processes (Mogoaşe et al. 2014; Hakamata et al. 2010) reported that AMT successfully reduces attention bias, anxiety symptoms and emotional vulnerability in both anxious and healthy individuals. Importantly, AMT training can increase attentional control among individuals high on anxiety (Cristea et al. 2015; Klumpp and Amir 2010), resulting in altered lateral frontal activation to emotional stimuli (Browning et al. 2010).

### Combining AMT with Non-invasive brain stimulation

Taken together, these studies suggest that attention training and neurostimulation have comparable effects in both affecting the neuronal activity of the DLPFC and modulating attentional biases towards emotional stimuli. Concerning the clinical efficacy of AMT, prior meta-analyses emphasized that the therapeutic benefit of the training is relatively small (Fodor et al. 2020; Mogoaşe et al. 2014), highlighting the importance of improving AMT paradigms or even combining it with treatments that will increase its effectiveness. As prolonged cTBS over the right DLPFC decreases the excitability of this cortical area, subsequent attention training might make use of this cerebral inhibition, resulting not merely in the accumulation, but in the improvement of the treatment effects.

The current study builds on previous research applying transcranial direct current stimulation (tDCS) over the DLPFC combined with attention training in participants at low or high risk for anxiety (Clarke et al. 2014; Heeren et al. 2015; Myruski et al. 2021). Findings suggested that tDCS stimulation over the left DLPFC combined with attention training reduces eye gaze duration on threatening stimuli compared to the group that received only the attention training among anxious individuals (Heeren et al. 2015). Clarke et al. (2014) used a similar design and provided evidence for significant changes in patterns of selective attention (e.g., decrease of attention bias to threat) for anxious participants receiving stimulation compared to those who received sham-stimulation. Additionally, Myruski et al. (2021) used bilateral tDCS (i.e., anodal in the left and cathodal in the right DLPFC) and found reduced attention bias to threat in a sample with low to moderate anxiety levels. Even though no significant changes in self-report anxiety were observed between the treatment groups, exploratory analyses showed that combined tDCS and AMT boosted stress resilience (Myruski et al. 2021). The current study explores the synergetic effects of neurostimulation and attention training using cTBS over the right DLPFC.

### Current study

The aim of the present study is to explore the effects of inhibitory cTBS over the right DLPFC combined with a computer-delivered attention modification training. In our efforts to improve the effectiveness of AMT on attentional control, both bottom-up and top-down sessions were included in the training. By combining these techniques, we aim to examine the synergetic effects of AMT and cTBS treatments. A randomized, sham-controlled design was applied, using eight sessions on consecutive days in a community sample of young adults differentiated on anxiety levels. Participants were randomly assigned to one of three groups where they would either receive (i) a combination of active cTBS and AMT, (ii) active cTBS and control condition of AMT, and (iii) sham cTBS with active AMT. In the sham condition of cTBS, a sham coil was used, which looks identical to its active version, replicates pulse noise, and mimics the sensation of magnetic stimulation. In this feasibility trial, we hypothesized that the combination of both treatments would amplify beneficial effects among anxious individuals by (1) decreasing anxiety symptoms, as assessed by self-report questionnaires, (2) decreasing attentional bias towards fearful faces and increasing attentional bias towards happy faces, as assessed by response times during a facial emotion dot-probe task, and finally (3) increasing eye gaze allocation and duration towards happy facial expressions, and especially the mouth region, as measured by an eye tracker device during a facial emotion dot-probe task. Mood questionnaires, attention bias, as well as eye gaze duration and direction were measured over two time points, before and after the treatment, in order to assess the effectiveness of the intervention.

We decided to include a comprehensive evaluation with multiple measures to better capture the effects of the treatment at a behavioral and attentional level. Attentional avoidance of threat (Calvo and Avero 2005; Garner et al. 2006; Rohner 2002), excessive attention towards threatening faces (Mogg et al. 2000; Rohner 2002) or lower eye-fixation times to positive stimuli (Chen et al. 2012) have been found by measuring eye gaze fixations in anxious participants. Hence, we decided to include an eye-tracking device which captures the dynamics of attention (Bendall et al. 2016) and might be a more valuable measure compared to traditional measures of reaction time (Chen et al. 2012). Further, eye tracking provides the opportunity to examine scan paths among certain Areas of Interest (AOIs) associated with emotional expressions, such as the eyes and mouth. For example, based on previous findings that showed longer eye gaze in the mouth region of happy faces, we expected to observe increases in attention specifically to the mouth region of happy facial expressions (Eisenbarth and Alpers 2011; Lischke et al. 2012). It is important to explore possible eye gaze changes between different facial regions after AMT and cTBS, similarly to Corman et al. (2020) who showed increased dwell time to positive information following the detection engagement trial of an attention training task. For all the analyses, gender was used as covariate, since females in non-clinical samples score higher on anxiety sensitivity (Armstrong and Khawaja 2002).

## Method

### Participants

Young adults between 18 and 25 years old were recruited via advertisements in the community and local universities for the purposes of the research project ‘*New Generation Interventions for Antisocial Behaviour: Transcranial Magnetic Stimulation combined with Attention Modification Training’*. All participants were Cypriots speaking Greek, who were selected from a larger community screening sample. Participants were informed that this is an “Innovative study using Transcranial Magnetic Stimulation in combination with computerized emotional training.” After treatment, we explained to participants that the study was designed to reduce their anxiety levels. Data collection took place in the two largest cities in Cyprus: Nicosia and Limassol. Depending on their place of residence, participants contacted a screening evaluation (Phase 1) at the Developmental Psychopathology Lab at the University of Cyprus, located in Nicosia, or at the Cyprus University of Technology, located in Limassol. For the current study, a sample of 89 individuals (*M*age = 21.29, *SD* = 2.06, 50.56% females) differentiated in anxiety levels participated in treatment programs (Phase 2). The study has been approved by the Cyprus National Bioethics Committee and informed consent procedures were followed.

**Exclusion criteria**. Based on the updated work of Rossi and colleagues (2020) for the safety and application guidelines of TMS in clinical practice and research, participants were screened for medical history and potential seizure threshold lowering factors (i.e., history of epilepsy/seizure, head trauma, brain surgery, tumor, intracranial metal implantation, migraines, medication use, sleep deprivation, infection, and alcohol consumption). Participants with active severe mental illness (e.g., psychosis), neurological disorders (i.e., Myasthenia Gravis) or receiving psychiatric medication were excluded from the study.

### Procedure

**Phase 1**. All participants provided informed consent and self-report questionnaires were completed through an online survey platform prior to the experimental assessments. The pre-treatment assessment lasted approximately 20 min and included one computerized task. Upon arrival in the lab, participants were instructed to seat opposite a computer screen (45 cm x 25 cm). Using portable *eye-tracker* equipment initial eye gaze and dwell time were monitored during the attention task. A calibration test was performed to check the accuracy of eye gaze recordings. Following calibration, participants completed a training phase with four pairs of the task in order to familiarize themselves with the procedure. Then, the visual dot-probe task was administered. Participants were instructed to indicate the location of the probe, which appeared after the presentation of emotional faces. After the attention task, the first phase of the study was completed.

**Phase 2**. Participants that met preliminary inclusion criteria continued with the treatment sessions of cTBS followed by AMT. The study was single-blind and sham-controlled. Participants were randomly assigned to one of the three different treatment groups: (i) cTBS and AMT, (ii) cTBS and control AMT, and (iii) sham cTBS and AMT. A short cTBS stimulation session (40s) over the right DLPFC was administered at the beginning of each session. After stimulation, participants were seated in front of a computer screen at an approximate distance of 80 cm and completed the control or the actual AMT. The duration of the treatment was eight consecutive daily sessions over a 2-week period, excluding weekends. Each session lasted approximately 20 min.

**Phase 3**. Post-treatment assessments were completed after the end of the treatment sessions. Each participant completed the same behavioral and attentional evaluation as descripted in Phase 1. At the end of the procedure, participants answered a questionnaire assessing treatment satisfaction. Finally, participants received a small financial compensation of 75 euros for their travel expenses.

### Self-report questionnaires

**Adult self-report inventory-4** (ASRI-4; Gadow et al. 2004). The adult self-report inventory includes behavioural symptoms of DSM psychiatric disorders. The ASRI-4 was completed by the participants using a 4-point Likert scale. For the current study, items corresponding to Generalized Anxiety Disorder (GAD; 8 items, a = 0.85) and Major Depressive Disorder (MDD; 11 items a = 0.82) were used. Research indicates that the levels of comorbidity between depression and anxiety is high among young adults, and we wanted to ensure that the treatment groups did not differ in both levels of depression and anxiety (Mahmoud et al. 2012). Studies involving community and clinical samples provided evidence for good reliability, convergent, and discriminant validity of the ASRI-4 scores (Gadow et al. 2004; Kyranides et al. 2017). Because we used a community sample very few participants met the Symptom Count Cutoff Score of ASRI-4, with the depression scale ranging from 1 to 30 and GAD from 2 to 24. For the purposes of the current study, we summed the anxiety scores to generate an aggregate symptom severity index based on T-Scores (Gadow et al. 2004). We considered scores lower or equal to 59 as low severity, 60–69 as moderate severity, and higher than 70 as high severity. Participants with moderate to high severity were considered at risk for anxiety.

### Experimental material

**Visual dot-probe task**. The visual dot-probe task is a well-validated method for assessing attentional bias (Bar-Haim et al. 2007) and is suitable for clinical research (Price et al. 2013). A central fixation cross (1000ms) was presented, followed by a pair of pictures briefly (500ms) displayed simultaneously side by side. One of the pictures was replaced by a probe (*). Participants were instructed to identify the probe as quickly as possible by pressing the corresponding arrow to indicate whether the probe appeared on the left or the right side. Facial expressions from the Karolinska Directed Emotional Faces (KDEF; see supplemental material 1; Lundqvist et al. 1998) dataset depicting fearful, happy and neutral faces were used. The KDEF is a widely used stimulus dataset and one of the most reliable systems for the experimental investigation of emotional processing (Goeleven et al. 2008).

The session consisted of 64 trials, divided in 2 blocks of emotional facial expressions, 32 trials per block. Face pairs were presented in one of the following potential parings: neutral-fearful and neutral-happy, following a randomized order to avoid sequential repetition of identical pairs. The probe appeared with equal frequency in the left or right side, as well as in the same (congruent) or opposite (incongruent) location as the emotional face. Following the most widely-used formula for bias score calculation (MacLeod et al. 1986), we computed a measure of attentional bias for threat by subtracting the subject’s mean Reaction Time (RT) to respond to probes that replaced faces displaying fear and happy emotions from the mean RT to respond to probes that replaced faces displaying neutral emotions. Increased scores on this measure indicate that either (a) attention was more readily oriented towards non-neutral items, which would speed responses to congruent trials, and/or (b) that disengagement of attention from non-neutral items was more difficult, which would slow responses to incongruent trials.

**Eye-tracking**. Participant’s eye gaze direction and duration were monitored during the dot-probe task described above via Tobii Pro Nano Eye Tracker (Tobii Technology, Sweden). Tobii Pro Nano is a standalone eye tracking equipment that uses infrared diodes to generate reflection patterns on the corneas of the user’s eyes, which are collected by image sensors. On each facial expression of the dot-probe task, two Areas of Interest (AOI) were created corresponding to the eyes and the mouth areas of facial expressions (e.g., fearful, happy and neutral). Using AOIs the following variables were examined (all measured in milliseconds): (1) Time to first fixation (i.e., the time corresponding to the first fixation for each AOI), (2) Total duration of fixation (i.e., the total time each participant fixated on each AOI) and (3) Number of fixations (i.e., the number of fixations occurring within each AOI).

**Continuous Theta Burst Stimulation (cTBS) Protocol**. For the current study, a figure-of-eight focal Air Film coil (AFC; 70 mm diameter) from Magstim Rapid^2^ was used and the methodological procedure was based on existing TMS guidelines (see Balconi and Canavesio 2013). According to the cTBS protocol, the power intensity was set at 80% of the active motor threshold (Huang et al. 2005) of each participant. A number of TBS studies used 80% of the active motor threshold over the prefrontal cortex (i.e., Cho et al. 2010; Cho et al. 2012; Ko et al. 2008; Ott et al. 2011), and according to a systematic review almost all studies that used 80% of active or resting motor threshold recorded significant stimulation effects (Ngetich et al. 2020). The theta frequency is defined as 5 Hz and cTBS is composed of triples; 3 pulses are given in a 50 Hz frequency. These 50 Hz triplets are repeated in a 5 Hz rhythm, and for cTBS burst 600 pulses are needed (3 pulses of 200 bursts). The stimulation lasted for 40 s consisting of one continuous cycle, which resulted in a neural inhibitory effect (Huang et al. 2005). The cTBS was applied over the right DLPFC. Using the international 10–20 positioning system, we identified F4 that corresponds to the right DLPFC stimulation target. The location and orientation of each participant’s coil placement were identified using an EEG cap. The coil was positioned with the handle pointing backward at a 45-degree angle between the coil handle and the nasion-inion line (midline) of the participant. In total, eight 40-seconds stimulations, one per day, over 2-week period were administered (excluding weekends).

#### Sham condition

A control condition using a sham coil was included in the experimental design to monitor the stimulation effect. An identical to the real Magstim figure-of-eight focal sham coil (70 mm diameter) was used, and participants experienced the same procedure as described above. Sham coil mimics both auditory and somatosensory side effects of TMS and studies show that the coil induces nearly zero electric-field under its center (Chistyakov et al. 2015; O’Reardon et al. 2007; Smith and Peterchev 2018).

**Attention Modification Training (AMT)**. AMT is a computer-delivered treatment that was used right after stimulation in order to reduce attention bias to threat and increase attention to positive stimuli. Based on published criteria (Bar-Haim et al. 2007), eight consecutive 20-minute sessions, over a 2-week period, were administered using the OpenSesame software (Mathôt et al. 2012). The training initially included four bottom-up sessions, which were followed by four top-down sessions (see the explanations below). The order was the same for all participants. All stimuli were extracted from the Radboud Faces Database (Langner et al. 2010), the International Affective Picture System (IAPS; Lang and Bradley 2007), and the Open Affective Standardized Image Set (OASIS; Kurdi et al. 2017). Three categories were selected: positive/happy, negative/angry and neutral.

*Bottom-up* sessions were based on modified versions of the dot-probe task in such a way that the probe nearly always (i.e., 80% of the trials; Hallion and Ruscio 2011) replaced the neutral and happy stimulus, thereby redirecting participants’ attention to non-threat cues (see supplemental material 1). The task had 300 trials, divided into five 60 trial blocks. Each trial began with a black dot presented in the center of a white screen for 500ms. Then, two faces of the same person appeared on the screen for 1000ms, one face on the top and one on the bottom, with equal distance from the screen center. Following the presentation of the faces, a probe (*) appeared in the location of one of the faces. Participants were instructed to select the location of the probe by pressing the corresponding arrow on the keyboard. The probe remained on the screen until a response was given. During each session, all the possible combinations of the probe position (top/down) and the face pairs (i.e., happy-neutral, angry-happy etc.) were presented in randomized order. For the *control* task, the same procedure and instruction was followed, but the probes appeared with equal probability across all stimuli. The face pairs (e.g., happy–neutral, angry–happy) were presented in a randomized order, with probes appearing across all stimuli types at equal frequencies. In order to gradually accustom the participant to the face tasks and keep variety among the sessions, different angles of the facial expressions were used. In particular, the following angles were selected: 1st session 90-degree angle where the actor looked directly at the participant, 2nd session 45-degree, 3rd 135-degree, and 4th session 0-degree where the face profile of the actor was visible (see supplemental material 1). This procedure followed suggestions that attention bias modification trainings need more captivating tasks (Mogoaşe et al. 2014), and we believed that variation in the experimental stimuli would enhance participant’s engagement.

*Top-down* sessions were based on visual search tasks (Corman et al. 2020; Pinkham et al. 2010), where participants were instructed to search for the positive face or picture and ignore other images in a 3 × 3 matrix. The task had 160 trials, divided into four 40 trial blocks, with 2 blocks for facial expressions and two blocks for pictures. Each trial began with a 500ms black dot in the center of a white screen. After the fixation point, 9 pictures were presented in random order in a 3 × 3 matrix and all pictures had the same size. For the facial expression blocks, each picture in the matrix represented a facial expression (i.e., happy, neutral and angry) of a different actor. The instruction was to click on the happy face using their computer mouse. The program continued to the next trial after a response was given. Only one happy facial expression appeared in each trial. Similar to the bottom-up session, the facial expressions had a different angle in each session. For the picture blocks, the participants were instructed to click on the positive stimuli (i.e., smiling baby) among other pictures (i.e., snake in an attacking position or a landscape) using their computer mouse (see supplemental material 1). Again, the participant proceeded to the next trial after a response was given. In the *control* task, all pictures were selected from the same database, and the same number of blocks and trials were used. However, the instruction was different. In the face blocks, participants were instructed to detect the neutral facial expression, and during the picture blocks individuals had to click on the neutral stimuli (e.g., a neutral facial expression or a neutral image like a galaxy) among the various pictures. This method was developed based on prior established paradigms (e.g., Waters et al. 2015).

**Plan of analysis**. Statistical analyses were performed using IBM SPSS Statistics software (Version 27). Data were screened for outliers as reported by the SPSS software; however, no extreme scores were identified. Prior to the main analyses, one way-Analysis of Variance (ANOVA) was performed to investigate significant differences among our treatment groups in age and mood disorders, while chi-square analysis was used for gender. In order to examine the effectiveness of the treatment, our main analyses included separate repeated measures ANOVAs for the questionnaire, behavioural and attentional data, similar to prior work (Konikkou et al. 2020; Li et al. 2022). First, possible treatment changes in anxiety were analysed by performing repeated measures ANOVA with self-reported anxiety pre- and post- treatment as the within subjects’ variable, with treatment (cTBS and AMT, cTBS and control AMT, and sham cTBS and AMT) and Anxiety (low and high risk) groups as between subjects’ variables. Second, to compare attentional bias scores across the emotional facial expressions (fearful and happy) from pre- to post-treatment measurements, repeated measures ANOVA was performed with treatment and Anxiety groups as between subjects’ variables. Finally, similar analyses were used to examine participant’s time to first fixation, total fixation duration and number of fixations in facial expressions before and after treatment. Separate repeated measures ANOVAs were conducted for each eye gaze measurement with treatment and Anxiety groups as the between subject’s variables. The two time points (pre- and post- treatment), the two predetermined facial areas of interest (eyes and mouth) and the three emotional expressions (fearful, happy, and neutral) were set as within subject’s variables. For all the repeated measures ANOVAs mentioned above, gender was used as a covariate. Finally, we mainly focus on time effects in order to evaluate the effectiveness of the treatment. Partial eta squares (Cohen 1988) and Cohen’s d effect sizes (Thalheimer and Cook 2002) are reported in the text.

## Results

### Descriptive statistics

Table 1 presents descriptive statistics for self-report measures assessing anxiety and depression, which were measured at Phase 1 of the current study. We proceeded with this analysis before treatment comparisons in order to examine possible group differences in internalizing problems, gender and age. The three treatment groups did not show significant differences regarding gender, age, and depression (see Table 1).

**Table 1:** Demographic information per group and groups’ differences on questionnaires before the treatment

	Sample	cTBS - AMT	cTBS – AMT control	sham cTBS - AMT	χ^2^	*p*
Total *N*	89	29	30	30		
Gender *N*					0.55	0.76
Males	44	13	16	15		
Females	45	16	14	15		

		M (SD)	M (SD)	M (SD)	F (2.84)	*p*
Age (years)		21.46 (2.30)	21.34 (2.13)	21.10 (1.81)	0.22	0.80
MDD		12.23 (4.71)	11.14 (6.45)	9.07 (5.58)	2.29	0.11

		M (SD)	M (SD)	M (SD)	F (2.84)	*p*
Anxiety	Control *N* = 40	7.00 (2.83)	7.00 (1.96)	7.14 (1.62)	1.32	0.24
Groups (GAD)	Anxious *N* = 45	14.60 (3.23)	16.19 (3.82)	13.56 (3.24)		

*Note*. *significant difference. GAD = Generalized Anxiety Disorder and MDD = Major Depressive Disorder as measured by Adult self-report inventory-4

*Anxiety groups*. Two groups were created during Phase 1 of the study: control (low severity, T-score ≤ 59) and Anxious (moderate and high severity, T-score ≥ 60), which were significantly different, *F*(1, 84) = 134.78, *p* <.001, in levels of anxiety. The rationale behind the two groups was to compare the efficiency of treatments for participants at low risk to those at moderate or high risk for anxiety. Additional differences in participants’ self-report anxiety were examined by taking into account the three treatment groups and the two Anxiety groups in an ANOVA, and this interaction effect resulted in non-significant differences (*F*(2, 84) = 1.32, *p* =.24; see Table 1). These findings indicate that within each anxiety group, participants in the treatment conditions did not differ in levels of anxiety. To test whether anxiety relates to the experimental measures, we run a correlational analysis. Findings suggested that self-reported anxiety was negatively correlated with attention bias to happy stimuli (*r* =-.25, *p* <.05), as well as the number of fixations to the mouth (*r* =-.23, *p* <.05) and eyes (*r* =-.28, *p* <.05) of happy faces. Therefore, increases in anxiety were associated with decreased attention to happy stimuli.

**Fig. 1:**
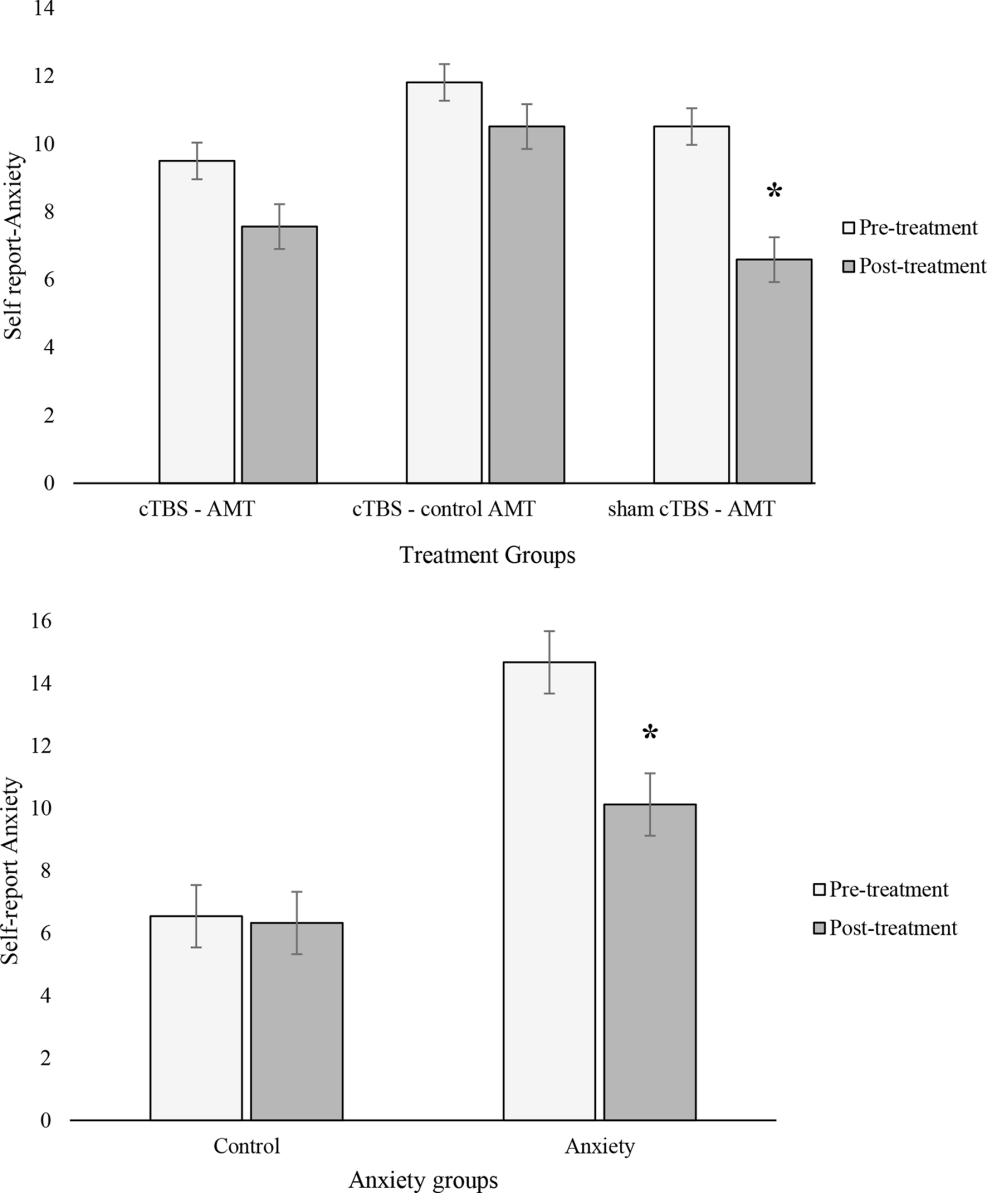
Interaction effects predicting Self-report anxiety pre-and post-treatment: Time x Treatment groups (first graph) and Time × Anxiety (second graph). *Note*. *significant differences pre and post treatment. Error bars indicate +/- 1 Standard Error

**Self-report Anxiety**. The repeated measures ANOVA examining differences in self-report anxiety prior and after treatment revealed three significant within groups effects. Firstly, in relation to **Time**, *F*(1,73) = 21.63, *p* <.001, η^2^ = 0.23, in general participants showed overall decreases in their self-report anxiety after treatment (*M* = 8.22, *SE* = 0.46, *p* <.001, *d* = 0.62) compared to their initial levels (*M* = 10.61, *SE* = 0.38; *p* <.01). Secondly, the **Treatment x Time** interaction, *F*(2,73) = 3.15, *p* <.05, η^2^ = 0.08, was significant. As we can see from fig. 1, all treatment groups showed a general decrease in self-report anxiety after receiving treatment, with the only significant effect identified for the sham cTBS-AMT condition with a high effect size (pre: *M* = 10.51, *SE* = 0.57; post: *M* = 6.59, *SE* = 0.70; *p* <.01; d = 1.15). Although pre to post differences in the other conditions were not significant, Cohen’s d effect sizes suggested moderate effects for the cTBS-AMT (pre: *M* = 9.50, *SE* = 0.82; post: *M* = 7.52, *SE* = 1.00; *d* = 0.42) and cTBS-AMT control (pre: *M* = 11.82, *SE* = 0.54; post: *M* = 10.51, *SE* = 0.66; *d* = 0.41) treatment conditions. Thus, AMT without stimulation might be more efficient in decreasing anxiety symptoms. The third within group result was an interaction between **Time and Anxiety groups**, *F*(1,73) = 17.88, *p* <.001, η^2^ = 0.20. Findings suggested that only anxious individuals showed a reduction in self-report anxiety after treatment (pre: *M* = 14.68, *SE* = 0.45; post: *M* = 10.12, *SE* = 0.56; *p* <.01; d = 1.35), whereas controls did not present significant changes over time, with small effect sizes (pre: *M* = 6.54, *SE* = 0.60; post: *M* = 6.32, *SE* = 0.74; *d* = 0.05; see fig. 1).

### Attention bias

Concerning the Attention Bias scores, the repeated measures ANOVA showed a significant interaction between **Emotions and Anxiety**, *F*(1,77) = 4.11, *p* <.05, *η*^*2*^ = 0.05. As expected, findings suggested that anxious individuals (*M* = 0.006, *SE* = 0.003) showed significantly higher attention bias to fearful facial expressions compared to those in the control group (*M*=-0.001, *SE* = 0.004, *p* <.05; d = 0.31), whilst the opposite finding was found for happy stimuli, with controls (*M* = 0.006, *SE* = 0.003) showing higher attention to happy stimuli compared to anxious participants (*M*=-0.001, *SE* = 0.003, *p* <.05; d = 0.30). These findings agree with the study’s proposed attention modification design.

**Fig. 2:**
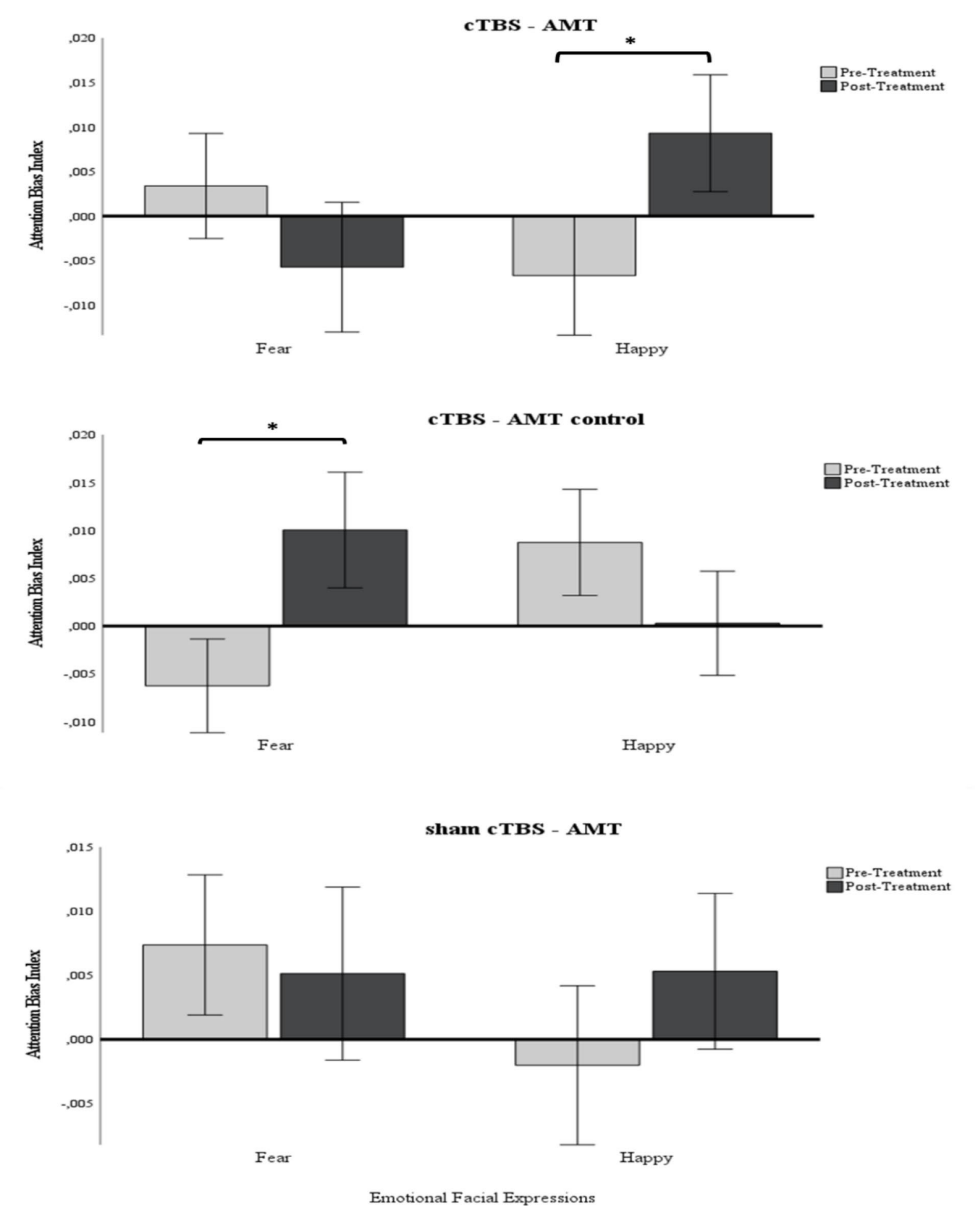
Pre and post treatment attention bias scores as measured by reaction times during a visual dot probe task for each treatment group and per each emotional facial expression (fear and happy). *Note*. *significant differences pre and post treatment. Error bars indicate +/- 1 Standard Error

The second significant interaction was **Time x Emotions x Treatment**, *F*(2,77) = 3.68, *p* <.05, *η*^*2*^ = 0.09. A significant increase of attention towards happy facial expressions was found for participants in the cTBS-AMT group after treatment (pre: *M*=-0.007, *SE* = 0.007; post: *M* = 0.009, *SE* = 0.007, *p* <.05; d = 0.43). However, the decrease in attention bias to fear stimuli for the cTBS-AMT group after treatment was not significant (see Fig. 2). In contrast, the cTBS- AMT control group showed increased attention bias only towards fearful faces after treatment (pre: *M*=-0.006, *SE* = 0.005; post: *M* = 0.01, *SE* = 0.006, *p* <.05; d = 0.54), indicating that cTBS alone is not efficient and might even have opposite effects. Finally, although participants in the sham cTBS-AMT treatment group showed similar changes in attention bias to both happy and fearful stimuli as the cTBS-AMT treatment group, these changes were not significant.

### Gaze data

**Time to first fixation**. Regarding the time that participants first fixated in an emotional face, neither the Treatment x Time interactions, nor the main effects of Time, Emotions, AOI or Groups were significant, all Fs < 3.025 and all ps > 0.120.

**Fig. 3:**
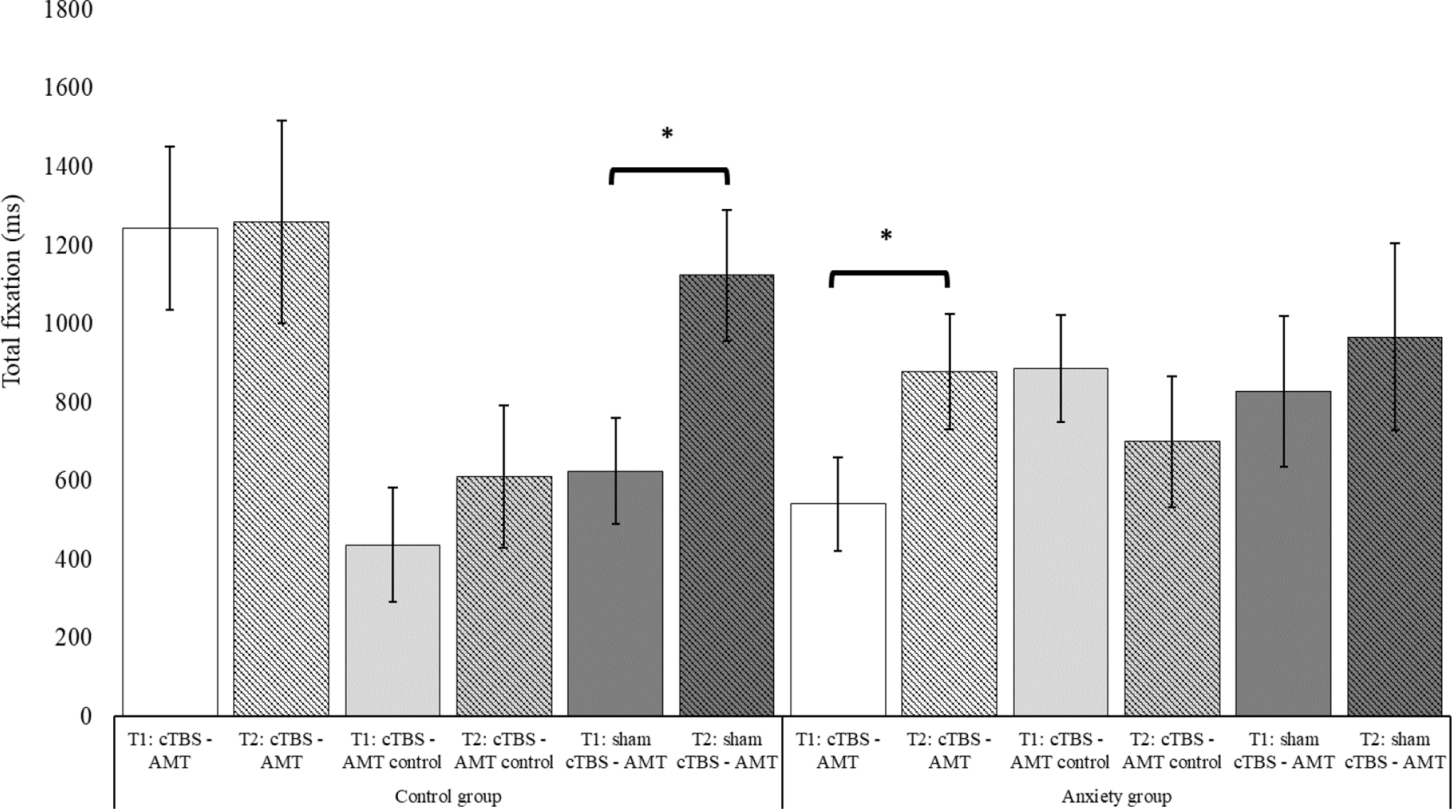
Time × Treatment × Anxiety group interaction for total time duration of fixation measured in milliseconds. *Note*. *significant differences pre and post treatment. Error bars indicate +/- 1 Standard Error

**Total duration of fixation**. Several important findings emerged from the repeated measures ANOVA in the total duration of eye gaze fixation. Firstly, a **main effect of the area of interest (AOI)** was identified, *F*(1,75) = 5.30, *p* <.05, η^2^ = 0.07, pointing to a general tendency for longer duration fixation in the eyes (*M* = 991.52, *SE* = 108.61) compared to the mouth region (*M* = 691.86, *SE* = 94.21, *p* =.05, *d* = 0.31) in our sample. Secondly, even though the Time x Treatment group interaction did not reach significance (*p* =.06), the three-way interaction **Time x Treatment x Anxiety** did, *F*(2,75) = 3.82, *p* <.05, *η*^*2*^ = 0.09 (see fig. 3). After breaking down the interaction in relation to *time* effects, control individuals showed a significant increase in the time of fixation in emotional stimuli after treatment only for the group that received sham CTBS and AMT (pre: *M* = 625.12, *SE* = 135.33; post: *M* = 1123.62, *SE* = 167.66; *p* <.01, *d* = 0.61). Individuals in the other two treatment groups scoring low on anxiety did not report significant within group changes before and after treatment. Concerning time effects among anxious individuals, again, significant results were identified only in one group, with significantly higher post treatment fixation time only among individuals in the combined cTBS-AMT group (pre: *M* = 540.86, *SE* = 119.24; post: *M* = 878.69, *SE* = 147.72; *p* <.001, *d* = 0.48).

**Fig. 4:**
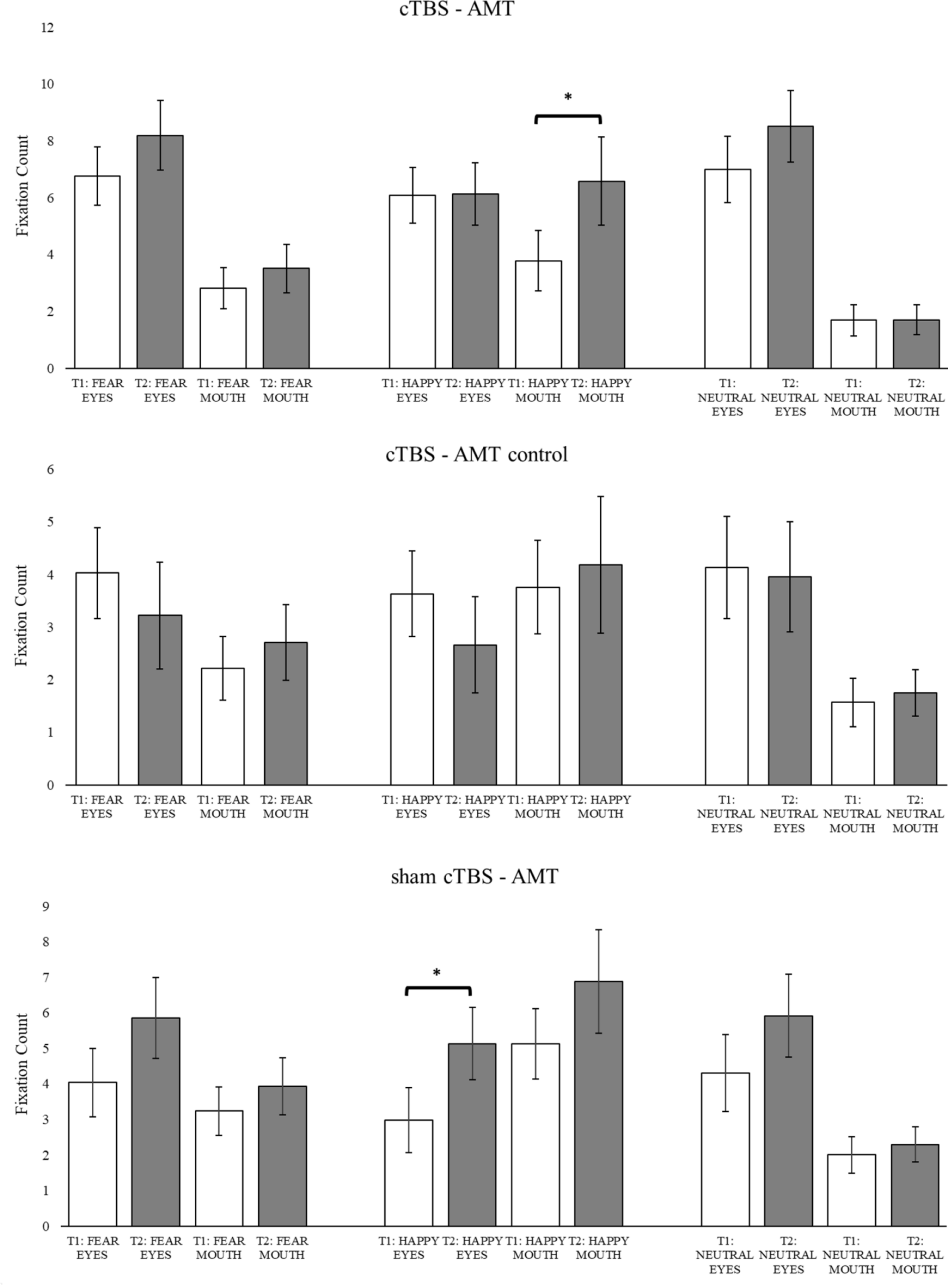
The interaction Treatment group × Time × Emotion × Area of Interest (AOI) predicting fixation count. *Note*. *significant differences pre and post treatment. Error bars indicate +/- 1 Standard Error

**Number of fixations**. The last eye gaze index focusses on the number of fixations within each area of interest (AOI) on the three emotional facial expressions. This particular measure indicated similar findings to the total fixation duration index, such as a significant **main effect of AOI**, *F*(1,78) = 9.95, *p* <.01, *η*^*2*^ = 0.11, with a higher number of fixations in the eyes (*M* = 5.33, *SE* = 0.54) compared to the mouth region (*M* = 3.08, *SE* = 0.41, *p* <.01, *d* = 0.50). Additionally, an interesting interaction was identified: **Treatment x Time x Emotions x AOI**, *F*(2,75) = 3.30, *p* <.05, *η*^*2*^ = 07. Examining *time effects* for each treatment group, a significant increase in the fixation counts pre to post treatment was identified in the mouth area of happy facial expressions only in the group that received both cTBS and AMT treatments (pre: *M* = 3.78, *SE* = 1.06; post: *M* = 6.59, *SE* = 1.56; *p* <.05, *d* = 0.40). The group that received only stimulation and a control condition of attention training, cTBS-AMT control, did not show any significant time difference across all emotions. The final group, sham cTBS-AMT, demonstrated an increase in fixation count prior to post treatment but in the eyes area of happy facial expressions (pre: *M* = 2.98, *SE* = 0.91; post: *M* = 5.14, *SE* = 1.02; *p* <.05, *d* = 0.41). These findings are illustrated in fig. 4.

## Discussion

The main aim of the present study was to investigate the possible synergetic action of combining a time efficient brain stimulation protocol over the right DLPFC with a computer-delivered Attention Modification Training on mood, attention bias and eye gaze metrices. Specifically, we aimed to examine the beneficial effects of cTBS and AMT separately on these multiple measures and, more importantly, to investigate whether combining both treatments may result in stronger effects, especially among participants with moderate to high levels of anxiety. Findings suggested that AMT with sham cTBS resulted in reduced self-reported anxiety symptoms; however, the beneficial effects of active cTBS with or without AMT only approached significance with a moderate effect size. Additionally, participants receiving the combined treatment protocol (cTBS-AMT) showed (1) increased attention to happy facial expressions, as suggested by behavioural data, (2) increased number of gaze fixations in the mouth region of happy facial expressions, and (3) higher fixation duration to emotional stimuli, which was mainly evident among anxious participants. Importantly, those who were in the stimulation-only condition did not show significant treatment effects, suggesting that it might be beneficial to administer AMT and cTBS together. These findings provide valuable information for future treatment programs designed to alter attention deficits, which combine novel neurostimulation methods, such as cTBS, and computer-delivered attention training.

### Anxiety symptoms

Findings in self-report anxiety indicated that anxious participants irrespective of treatment showed decreases in anxiety, with those receiving AMT-only showing the higher decrease in anxiety symptoms after treatment. In contrast to several literature reviews emphasizing on the clinical weakness and the lack of validity of attention training procedures (Cristea et al. 2015; Dennis-Tiwary et al. 2019), our findings confirm the efficacy of AMT in reducing self-report anxiety levels (Amir et al. 2009; Hakamata et al. 2010; Mogoaşe et al. 2014; Schmidt et al. 2009). More importantly, the beneficial effects of the training are amplified in anxious populations, which agrees with prior work showing positive effects of the training on patients with generalized anxiety disorder (Mogoaşe et al. 2014). Together, these findings confirm a beneficial effect of AMT in anxiety symptoms, replicating previous findings in both adult and pediatric populations across various settings (i.e., laboratory, school, and home) (Dandeneau et al. 2007; De Voogd et al. 2014; Waters et al. 2016).

Despite the promising effects of AMT, it is also important to mention that this reduction in self-report anxiety was evident in both the cTBS-AMT and cTBS-AMT control groups; however, the reduction in anxiety among participants receiving sham cTBS and AMT resulted in significant and stronger effects. Other studies also observed this phenomenon (see review by Trevizol et al. 2016), and a possible explanation of this finding is that by targeting the right DLPFC we affected specific cognitive processes that might not be related to anxiety symptoms. According to the review of Ngetich and colleagues (2020), cTBS over the right DLPFC impaired attention, inhibitory control, planning, and goal-directed behaviour in decision making but also improved decision making by reducing impulsivity. Thus, possible changes in cognitive functions resulting from cTBS treatment might not be captured by a behavioural mood questionnaire and require measurements using an attentional task, to identify specific mechanisms of change.

In our effort to improve its clinical performance, we created two aspects of AMT trials, bottom-up and top-down sessions, aiming to direct participants attention to positive stimuli using both automatic and cognitive controlled mechanisms. In line with our AMT, prior work suggested that such approach-faces training in anxious individuals led to more positive mood and reduced anxiety instead of training that focused on avoiding emotional faces (Rinck et al. 2013). Our approach agrees with the suggestion that multisession AMT-positive-search training might be a promising intervention for reducing anxiety symptoms (Mogg et al. 2017; Dandeneau et al. 2007; De Voogd et al. 2014; Waters et al. 2016). According to theoretical frameworks, anxious individuals pay less attention to positive stimuli, which also agreed with our pre-treatment findings (Mogg et al. 1995). Therefore, by increasing attention allocation towards positive stimuli using attention training, we could target anxiety symptoms and enhance positive feelings.

### Changes in attention bias

In the present study there was a significant finding suggesting that participants in the cTBS-AMT group showed faster detection of the probe following happy faces after treatment. This finding points to a beneficial effect of cTBS combined with AMT in inhibitory cognitive control processes, resulting in increased attention to positive information. Moreover, participants in the cTBS-AMT group showed increased number of gaze fixation in the mouth region of happy facial expressions. Looking at the mouth area contributes to the recognition of happiness (Beaudry et al. 2014) and numerous studies highlight the importance of the smiling mouth for a happy facial expression than the eyes (Calvo et al. 2014; Eisenbarth and Alpers 2011; Lischke et al. 2012). Finally, the cTBS-AMT group increased their fixation duration to emotional stimuli after treatment sessions, which was stronger for anxious individuals. The current study showed a clear shift of attention when neurostimulation and attention training were combined. This finding is in line with previous work, which applied transcranial direct current stimulation over the DLPFC combined with attention training among anxious participants (Clarke et al. 2014; Heeren et al. 2015; Myruski et al. 2021). Thus, the participants receiving the combined treatment learned to reallocate their attention and to actively search for positive information, providing support for the combination of neuro-stimulation and attention oriented treatments.

In the present study, no evidence of decreased attention to fearful faces was found. The aim of AMT in our study was to increase attention to positive stimuli, and previous studies established that the processing of negative and positive information are two different mechanisms (Garland et al. 2010; Noguchi et al. 2006). During the AMT sessions in our study the probe directed participants’ attention towards the smile area of a happy face (bottom-up session), or the participants were instructed to find the happy face among arrays of emotional faces (top-down sessions), following suggestions that the processing of facial expressions involves both bottom-up and top-down flow of information (Hadders-Algra 2022). Thus, the goal of the current study was to create a training that directs attention, using automatic and goal driven processes, into the smile area of a happy face and to combine this training with cTBS over the right DLPFC, which improves decision making functions (Ngetich et al. 2020). It is generally observed that low anxious individuals exhibit increased attention toward positive stimuli (i.e., happy faces) compared to neutral stimuli (Liang et al. 2017), whereas this tendency is less prominent and occasionally reversed in high anxiety (see review by Frewen et al. 2008). Our findings agree with the results by Corman and collaborators (2020), who found that the inclusion of a visual detection search AMT mainly resulted in enhanced positive attentional bias. Such visual search paradigms seem to be effective particularly in community samples where a pre-treatment attentional bias to threat is not always present (Eldar et al. 2008). It was interesting that even participants in the control group in our study showed increased fixation duration to emotional stimuli after attention modification.

Additionally, DLPFC inhibition has been associated with increased activity in response to happy emotional faces (Cao et al. 2018; Konikkou et al. 2020), suggesting an important role of this particular brain region in emotional processing. However, in our study, the right DLPFC theta burst stimulation alone was not sufficient to result in eye gaze alterations during emotional faces as measured by eye-tracking time and visit counts; while the opposite was found for cTBS and control AMT. In particular, the stimulation-only group (cTBS-control AMT), showed increased attention bias towards fearful faces. This finding is not surprising in the literature. For example, after inhibitory stimulation of the right DLPFC participants showed a stronger orienting response towards angry faces (d’Alfonso et al. 2000). Therefore, additional research is needed to evaluate the effects of cTBS-only over the right DLPFC in attentional processes. Nevertheless, current mechanistic findings provide valuable information regarding the effectiveness of combined AMT with cTBS protocols over the right DLPFC to increase attention towards positive information.

### Limitations, strengths, future directions and implications

Current results must be considered in light of some study limitations. The participants of the present study were anxious individuals with moderate to high anxiety and not clinically diagnosed for anxiety. This paves the way for further investigation into the applied value of such combined treatments among individuals with clinical levels of anxiety. Moreover, even though we used a randomization procedure, the current study design does not account for participants’ expectations. There are several studies positing that sham electrical and magnetic stimulation is able to induce an effect in different cognitive and motor domains, likely because of expectations (for a review, see Braga et al. 2021). We agree that the ideal design to fully isolate the effect of expectation related to the coil placement would include a fourth group receiving AMT only, without any type of stimulation. However, our study was designed to investigate the added benefit of cTBS to AMT, rather than to specifically examine the influence of expectation. The comparison of cTBS + AMT to sham cTBS + AMT group directly addresses this question, allowing us to assess whether the active stimulation provides a greater effect than what is observed with the sham procedure, which inherently includes a degree of expectation. While we acknowledge that the sham group might overestimate the general placebo effect due to the coil placement, this limitation does not detract from our primary research question, which is focused on the relative effectiveness of cTBS + AMT compared to sham + AMT. Future research could benefit from a direct comparison of sham stimulation with a no-stimulation control group to fully disentangle the effects of expectation. Further, it is highly recommended that future work incorporates the use of a neuronavigation system to precisely place the coil to stimulate the target brain area and consequently magnify the accuracy and robustness of the stimulation effects. Moreover, future studies could use variations of AMT for assessment purposes, such as the visual search paradigm, with longer presentation of emotional stimuli or add virtual reality settings and account for possible eye gaze changes to examine real world transfer of the training.

Strengths of the present investigation include the multi-method assessment, which effectively explored attentional biases changes after treatment by combining dot-probe assessments with eye gaze measurements. In this study, eye gaze metrics formulated a better understanding of how each treatment works, highlighting the importance of a comprehensive evaluation with multiple measures to better capture the effects of the treatment at a behavioural and attentional level. At the same time, we were able to examine anxiety changes using self-report questionnaires. In addition, the methodological design not only used a sham cTBS coil for control conditioning of stimulation, but also we added a control training specifically designed to match the AMT training. Another strength of the current study is the use of multiple AMT sessions for altering attentional bias that included both bottom-up and top-down attentional processes. Present findings are ideally suited for prioritizing the proposed stratification in future work, informing individualized treatments.

The findings of this study suggested that by stimulating brain areas associated with attention deficits, cTBS can enhance the effectiveness of AMT. Indeed, Transcranial Magnetic Stimulation has shown great promise in the literature, and it is becoming an increasingly popular therapeutic tool because it allows for the direct manipulation of neural networks. The US Food and Drug Administration (FDA) has cleared the use of intermittent Theta Burst Stimulation to provide treatment to patients with major depressive disorder (Brooks and Clears 2018), and our study suggests that such protocols should be extended to anxiety. The identification of such clinical effects of different protocols, as is the case for the present study using a time efficient – cTBS in anxiety, can be used to minimize the adverse effects of pharmacotherapy. For instance, TMS was used in medication-resistance depression patients (Perera et al. 2016), and cTBS over the right hemisphere is able to influence the response to medication among patients with depression (Ngetich et al. 2020). As suggested by Konikkou et al. (2020), understanding the effects of cTBS will provide the opportunity for clinicians to choose the most appropriate protocol according to the individual needs of each patient (i.e., excitatory or inhibitory stimulation). We are aware that the 8-consecutive neurostimulation sessions used in the current study may not result in a long-lasting brain plasticity effect, since the traditional TMS treatment protocols include 30 consecutive sessions (O’Reardon et al. 2007). However, our results suggest a promising pathway for the synergetic action of cTBS and AMT. Therefore, additional cTBS sessions may lead to even higher reductions in anxiety symptoms.

AMT, and particularly the visual search for a positive paradigm, showed beneficial effects on targeting anxiety symptoms. AMT is an emerging technique that can be used as a psychotherapeutic application on mobile and phone-based technologies in general (e.g., Myruski et al. 2021). Thus, although AMT is advised to be administered initially in laboratory conditions for better clinical efficacy (see Mogoaşe et al. 2014), it might later result in an additional tool for an alternative no-talk therapy. Personalized cues that trigger emotional responses, such as familiar faces and expressions can also be used in future study designs to enhance the participant’s emotional experience and engagement. Also, the AMT sessions could be customized in accordance with the attentional mechanism and deficits of the anxious patient, such as attention bias to negative stimuli, insensitivity to positive or attentional avoidance of threatening stimuli. For example, AMT where participants attention is directed to the threatening stimulus may help to reduce attentional avoidance; an exposure condition to the disorder-related stimuli. On the other hand, AMT where participants attention is directed to the positive stimulus may help to reduce attention bias toward negative stimuli.

In conclusion, current mechanistic findings suggest that AMT is a promising tool to target anxiety symptoms. More importantly, when AMT is combined with inhibitory stimulation over the right DLPFC results in increased eye gaze duration and allocation towards smiley facial expressions among anxious individuals, leading to increased attention towards positive information. These clinical indications point to the importance of using multidimensional treatment models to maximize clinical efficiency, including brain activity techniques and attentional interventions. Although additional evidence is needed for its clinical efficacy, present findings contribute to our understanding of inhibitory stimulation over the right DLPFC combined with AMT on emotion processing can decrease anxiety related symptoms. By increasing such knowledge, we can inform novel ways of targeting neural responses associated with mood and attentional control.

## Electronic supplementary material

Below is the link to the electronic supplementary material.

Supplementary Material 1

## Data Availability

No datasets were generated or analysed during the current study.
