# Supplementary material for: Testing the effectiveness of combined attention modification training with right dorso-lateral prefrontal cortex theta-burst stimulation on reducing levels of anxiety and attentional bias
Source: Exp Brain Res. 2025 May 4;243(6):137. doi: 10.1007/s00221-025-07040-9 (PMC12050235; doi:10.1007/s00221-025-07040-9)

**Supplemental material 1**

*Attention Modification Training. Bottom-up sessions were based on modified versions of the dot-probe task in such a way that the probe nearly always replaces the neutral and happy stimulus by directing participants to the mouth region of the facial expression. Top-down sessions were based on previous visual search tasks - where participants were instructed to search for the positive face or picture and ignore other images.*

**
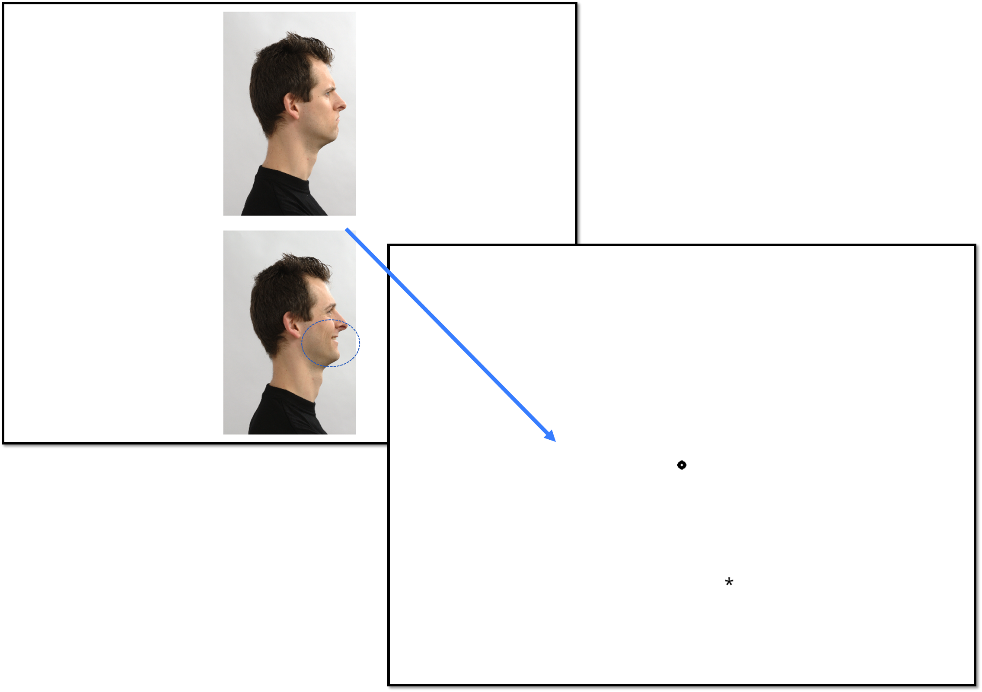
**

Bottom-up sessions

Top-down sessions


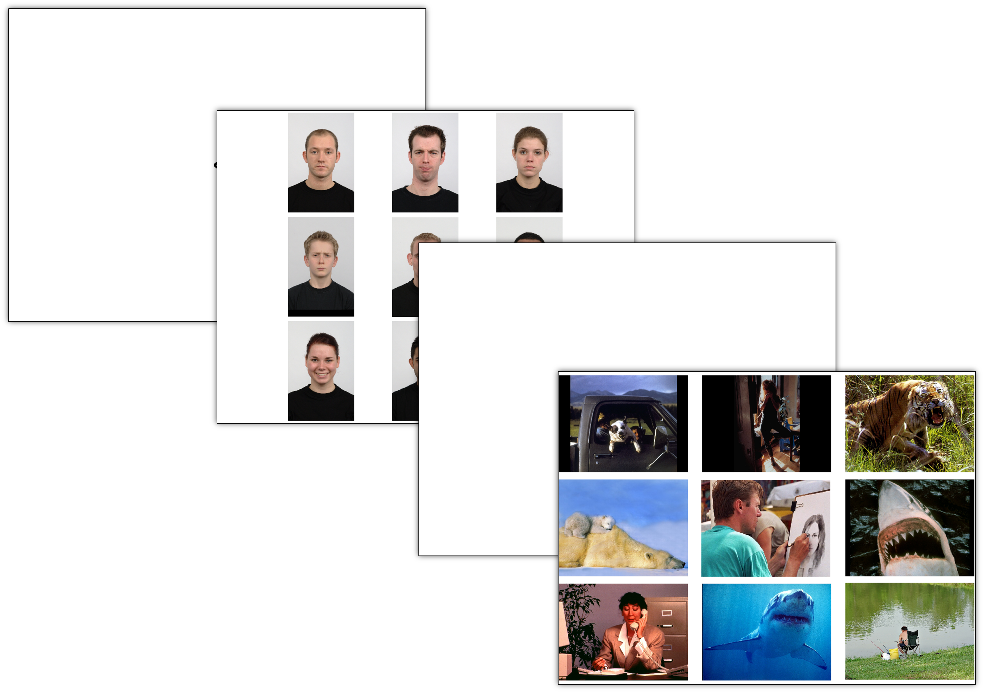


*Happy facial expressions in different angles retrieved from Radboud Faces Database (Langner et al., 2010) for the Attention Modification Training. Starting from left 0-degree. 45-degree. 90-degree and 135-degree.*


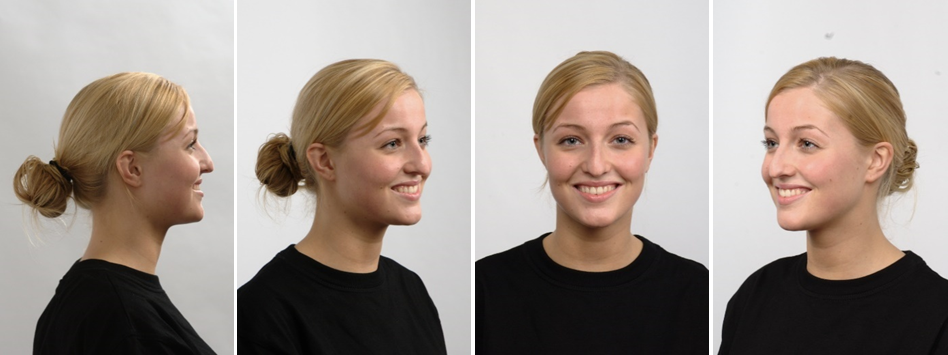

Supplement: Supplementary file 1 — Supplementary Material 1 [file 221_2025_7040_MOESM1_ESM.docx]
